# Monocular reconstruction of shapes of natural objects from orthographic and perspective images

**DOI:** 10.3389/fnins.2024.1265966

**Published:** 2024-04-15

**Authors:** Mark Beers, Zygmunt Pizlo

**Affiliations:** Department of Cognitive Sciences, University of California, Irvine, Irvine, CA, United States

**Keywords:** inverse problems, monocular 3D vision, shape reconstruction, symmetry, compactness

## Abstract

Human subjects were tested in perception of shapes of 3D objects. The subjects reconstructed 3D shapes by viewing orthographic and perspective images. Perception of natural shapes was very close to veridical and was clearly better than perception of random symmetrical polyhedra. Viewing perspective images led to only slightly better performance than viewing orthographic images. In order to account for subjects’ performance, we elaborated the previous computational models of 3D shape reconstruction. The previous models used as constraints mirror-symmetry and 3D compactness. The critical additional constraint was the use of a secondary mirror-symmetry that exists in most natural shapes. It is known that two planes of mirror symmetry are sufficient for a unique and veridical shape reconstruction. We also generalized the model so that it applies to both orthographic and perspective images. The results of our experiment suggest that the human visual system uses two planes of symmetry in addition to two forms of 3D compactness. Performance of the new model was highly correlated with subjects’ performance with both orthographic and perspective images, which supports the claim that the most important 3D shape constraints that are used by the human visual system have been identified.

## Introduction

Perception of three-dimensional (3D) shapes from a single two-dimensional (2D) image is one of the most difficult problems in vision science, a problem which remains unsolved both in human and computer vision. 3D vision is an ill-posed inverse problem (Pizlo, 2001). The ill-posedness is caused by the fact that the depth dimension is lost in the 2D image and must be recovered. Any given 2D image could be a projection of infinitely many 3D objects. So, when provided a single image, the human visual system is confronted with great ambiguity regarding the 3D scene which generated the image. Yet, our everyday informal observations suggest that monocular perception of real objects is often veridical. By veridical, we mean that we see 3D shapes the way they are “out there.” Mathematics dictates that the only way to arrive at a unique, let alone veridical, 3D interpretation of a 2D image is to impose constraints (Poggio et al., 1985). Therefore, the central question is to identify the constraints the human visual system employs to achieve unique and often veridical reconstruction of natural objects.

Despite the importance of natural objects, much of the past work on 3D perception has only considered very simple objects. Hochberg and McAlister (1953) explored under what viewing directions Necker cubes were perceived as 2D or 3D. Necker cubes are always perceived as 3D unless viewed from very specific directions, where the 2D image of the Necker cube is very simple. Their work and much subsequent work emphasized the role of a simplicity principle, where in the presence of ambiguity, the ‘simplest’ interpretation of the 2D image is the one selected by a subject or model.

Other work has emphasized the role of rectangularity as a constraint in 3D monocular perception. Man-made objects such as bookshelves and tables often have right angles. Is it the rectangularity that is responsible for veridical perception of shapes of natural objects? Perkins (1972, 1976) considered images of opaque, deformed, box-like objects, and found that subjects indeed often perceived 3D interpretations with right angles when the 2D image admitted such interpretations. Rectangularity is likely a constraint used by the visual system, either explicitly or implicitly.

Perkins’ work provides a good example of an experimental technique used to query the 3D percept of a subject. Namely, subjects are asked to report some feature of their 3D percept. Perkins (1976) asked subjects to report a perceived 3D angle. Attneave and Frost (1969) provided images of rectangular stimuli and asked subjects to adjust the 3D slant of an edge. Other techniques for investigating a subject’s 3D percept exist. Shepard and Metzler (1971) performed an experiment in which two images were presented to a subject, where the two images were of the same object from a different viewpoint, or of different objects. If the subject correctly identified that two different images were of the same object, the subject achieved shape constancy. By shape constancy we mean the same 3D interpretation is achieved despite different 2D views. Human shape constancy is reliable with symmetrical, regular objects (Biederman and Gerhardstein, 1993; Li et al., 2011) but poor with highly unstructured objects (Rock and DiVita, 1987). Shape constancy experiments enable the experimenter to identify conditions where 3D shapes are perceived reliably, but do not give the experimenter access to the subject’s 3D percept. For this, we need shape reconstruction experiments. In shape reconstruction experiments, a subject is provided a static image of a 3D shape and a set of 3D interpretations of that image. The subject’s task is to identify which 3D shape in the provided set they perceive while looking at the 2D image.

Suppose we have access to a subject’s 3D percept via a shape reconstruction experiment. The goal is to formulate a computational model that generates the same 3D reconstruction as a human subject given the same 2D information. What constraints other than rectangularity could be used as an implementation of a simplicity principle? If an object is “simple” perhaps its 3D angles ought to be similar. Marill (1991) proposed a model which selected a 3D reconstruction with minimal standard deviation of angles (MSDA). This model was able to recover polyhedral shapes to high degrees of accuracy. Leclerc and Fischler (1992) showed that combining MSDA with planarity produced even more accurate 3D interpretations. Biological shapes, such as animal bodies, would seem smooth enough that rectangularity, MSDA and planarity no longer function as good constraints.

Symmetry has been proposed as a more general alternative, and is clearly a version of a simplicity principle. Mirror symmetry has been used to achieve good reconstruction results on simple, often polyhedral objects (Vetter and Poggio, 1994; Sawada, 2010; Li et al., 2011; Jayadevan et al., 2018). Many of these models also incorporate a compactness constraint, which maximizes volume of a 3D recovered shape for a given surface area. Until now, none of these models have been tested extensively on natural objects.

In nearly all of the cited experiments, orthographic approximations to perspective images were used. In making the transition from simple objects to natural objects, it makes sense to investigate what impact this approximation has on 3D perception. One of us showed that the visual system produces more reliable percepts of 2D slanted shapes when an orthographic approximation to a perspective projection is used (Sawada and Pizlo, 2008). Will this advantage translate to 3D shapes?

First, we describe our psychophysical experiment on 3D shape reconstruction. Then, we describe a new computational model and compare the model performance to the performance of the subjects.

## Psychophysical experiment

### Methods

#### Subjects

Three subjects, including one author (S1) participated in this experiment. All subjects had normal or corrected-to-normal vision. Subject S1 received extensive practice before data collection. Subject S3 was naïve with respect to the hypotheses being tested.

#### Stimuli

**Figure 1:**
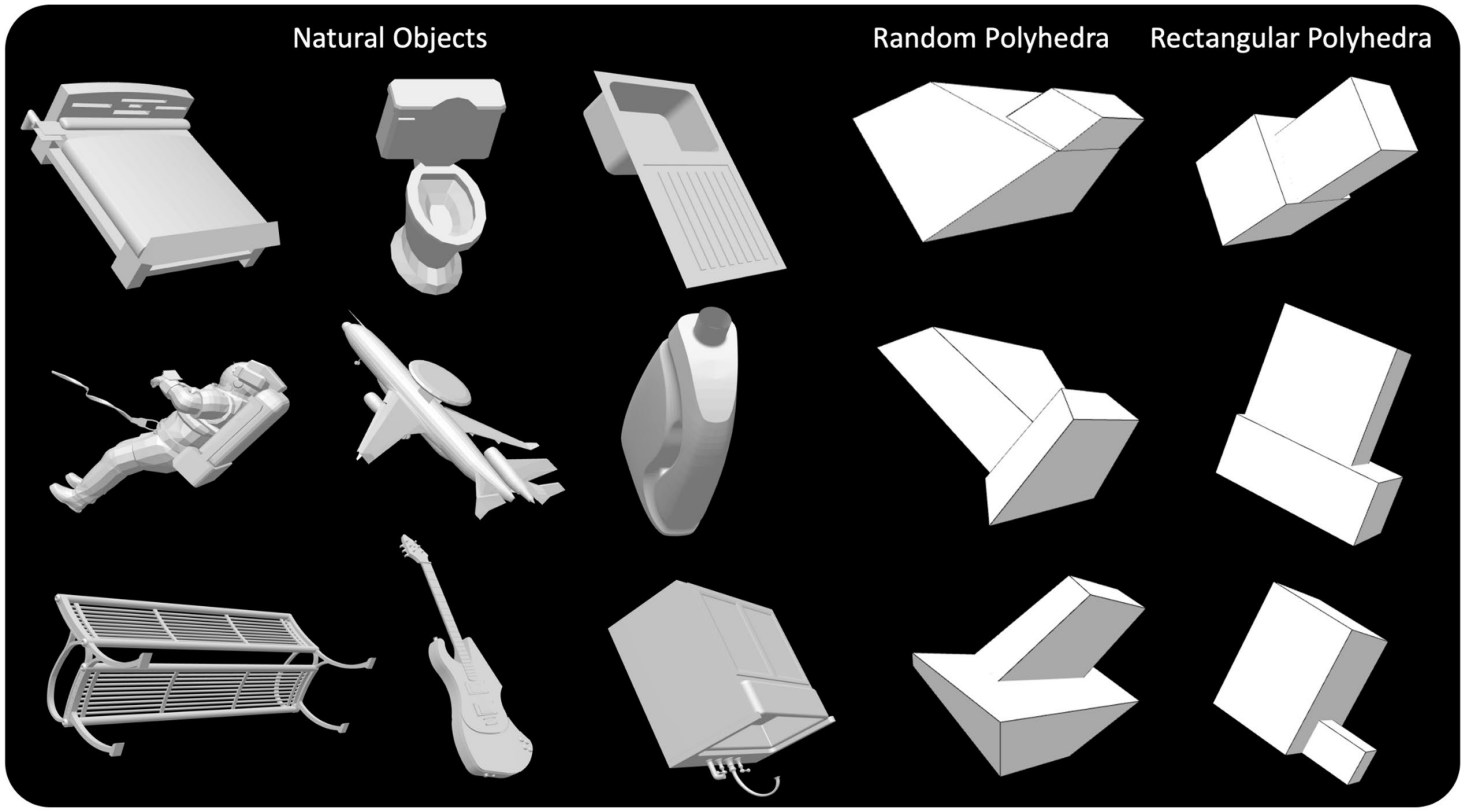
Perspective images of natural objects, random symmetrical polyhedra, and rectangular symmetrical polyhedra.

Three types of objects were used (see examples in Figure 1). First, natural objects (cars, airplanes, chairs, beds, desks, etc.) were selected from the ModelNet40 dataset (Wu et al., 2015). These natural objects were selected to have one and only one plane of symmetry which accounted for the vast majority of points in the mesh defining the object. These objects may have contained parts with more than one plane of symmetry. For example, a bed may have one global plane of symmetry but the mattress atop the bed is a rectangular prism with three planes of symmetry. Many of the symmetrical objects in the ModelNet40 dataset are aligned such that their primary symmetry plane is roughly parallel to the XY, XZ, or YZ plane. Regardless, we rotated and translated all these 3D objects such that their primary symmetry plane was coplanar with the YZ plane. In our coordinate system, *Z*-axis represents the depth dimension, *X*-axis is horizontal and Y axis is vertical.

Second, random symmetrical polyhedral objects were generated, similar to those used in our prior experiments (Li et al., 2011; Jayadevan et al., 2018). These objects were symmetrical about the YZ plane, had planar faces, and their aspect ratios did not exceed nine. These objects were composed of two appended boxes, such that the bottom faces of the two boxes were coplanar. These random polyhedra were constructed such that no angle on a face of a random polyhedron was 90 degrees, and 95% of these angles differed from 90 deg. by more than five degrees. The relative sizes of the two boxes composing a random polyhedron varied over a wide range.

Third, rectangular symmetrical polyhedral objects were generated. As pointed out in the Introduction, rectangular polyhedra were used to evaluate rectangularity as a possible *a priori* constraint. These objects were composed of two appended rectangular prisms, such that the bottom faces of the two rectangular prisms were coplanar. The rectangular polyhedra were generated such that no aspect ratio could exceed nine. The rectangular polyhedral objects were also mirror symmetrical about the YZ plane. The relative sizes of the two boxes composing a rectangular polyhedron also varied over a wide range.

Despite the fact that rectangular polyhedra had 90 deg. angles and random polyhedra did not have 90 deg. angles, they all were generated in a similar way. In both cases, a cross section of an object along its symmetry plane was defined first. In the random polyhedra case, this looked like two convex quadrilaterals appended. In the rectangular polyhedra case, this looked like two rectangles appended. Edge lengths in cross sections were sampled from the same distribution in both rectangular polyhedra and random polyhedra cases. Then, widths of each box were sampled from the same distribution for both random polyhedra and rectangular polyhedra. In random polyhedra, three widths were sampled for each box and the fourth width was picked to ensure that faces of the polyhedron were planar. If this fourth width from planarity was too large or caused intersections, new widths were sampled. Because the edge lengths of the rectangular polyhedra and random polyhedra were sampled from the same distribution, the distribution of aspect ratios of the 3D shapes was similar.

### Procedure

The subject performed the experiment monocularly in a dark room. The subject’s head was supported by a chin-forehead rest 20 inches from a computer monitor (32″ diagonal, 137 pixels per inch). The line connecting the subject’s uncovered eye with the center of the monitor was orthogonal to the surface of the monitor.

**Figure 2:**
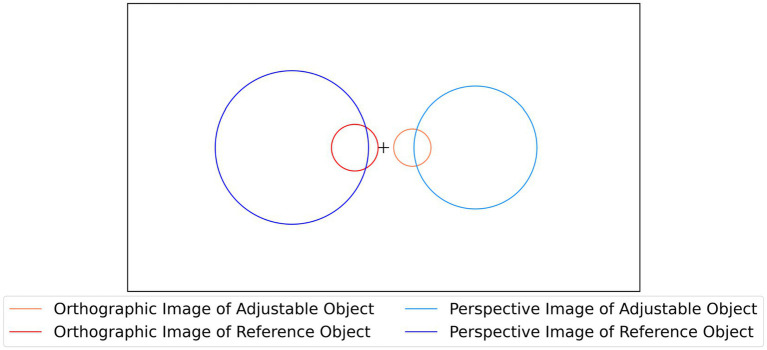
The black rectangle represents the boundary of the computer canvas, while the circles represent average image sizes and positions during the experiment with perspective and orthographic projection. Images were placed at ±4.5 degrees of visual angle from the center in the orthographic case and ±14 degrees of visual angle from the center in the perspective case. On average, the perspective image of the reference object occupied 22.8 degrees of visual angle, while the perspective image of the adjustable object occupied 18.5 degrees of visual angle. On average, the orthographic image of the reference object occupied 7.2 degrees of visual angle, while the orthographic image of the adjustable object occupied 5.8 degrees of visual angle.

Six different conditions were used, for each combination of object type (natural objects, random polyhedra, rectangular polyhedra) and projection type (orthographic, perspective). In each trial of the experiment, the subject was shown a 2D image of a stationary *reference shape* and a rotating, symmetrical, *adjustable 3D shape* consistent with an orthographic image of the reference shape. The subject adjusted the aspect ratio of the rotating 3D shape until it matched the 3D percept produced by the 2D image of the reference shape. Across all conditions, no 3D shape was presented more than once. All three subjects were tested on the same shapes. This allowed computing correlations between pairs of subjects in all 6 experimental conditions. The position and size of images and reconstructions during each trial of this experiment are described in Figure 2.

Consider first the orthographic projection. It is known that a 2D orthographic image of a 3D mirror-symmetrical shape does not allow for a unique reconstruction of the 3D shape (Vetter and Poggio, 1994; Li et al., 2011). Specifically, the 2D orthographic image determines the 3D symmetrical shape up to a single free parameter. This parameter is the slant angle of the symmetry plane of the 3D reconstructed shape. Changing the slant of the symmetry plane of the 3D reconstructed shape leads to changes of the aspect ratio of the 3D shape [see Eq. B4 in Li et al. (2011)]. The relation between the slant and the aspect ratio is one-to-one. On each trial, the subject used two keys on a keyboard to change the aspect ratio of the adjustable 3D shape. The discrete steps of this adjustment corresponded to changes of the slant of the symmetry plane by 0.01 radians per key press. The initial aspect ratio of the adjustable 3D shape was random, and it corresponded to a slant angle between 0.2 and π/2–0.2.

Next, consider the perspective projection. It is known that a 2D perspective image of a 3D mirror-symmetrical shape allows for unique reconstruction of the 3D shape (Sawada et al., 2011). So, geometrically, reconstruction from perspective images is easier than from orthographic images. Will this geometrical fact lead to a more accurate percept of a 3D shape? The most direct way to answer this question is to use the same one-parameter family of 3D shapes in the adjustment and verify whether there was an improvement in reconstructed 3D shapes. Perspective images on the computer monitor were produced by placing the center of perspective projection at the subject’s eye. This means that the retinal images produced by the perspective images on the computer monitor were correct perspective images of the simulated 3D shapes. Similarly to what was done in the session that used an orthographic projection, the adjustable 3D shape was always a member of the one-parameter family of 3D shapes consistent with an orthographic image of the reference 3D shape. Note that the reference 3D shape was the only member of the one parameter family of 3D shapes that could geometrically match the stationary perspective image shown on the left, so the perspective projection played a role of a constraint in the shape adjustment. This suggests that subject’s reconstruction of the 3D shape in perspective projection conditions could be more accurate compared to the orthographic projection conditions.

**Figure 3:**
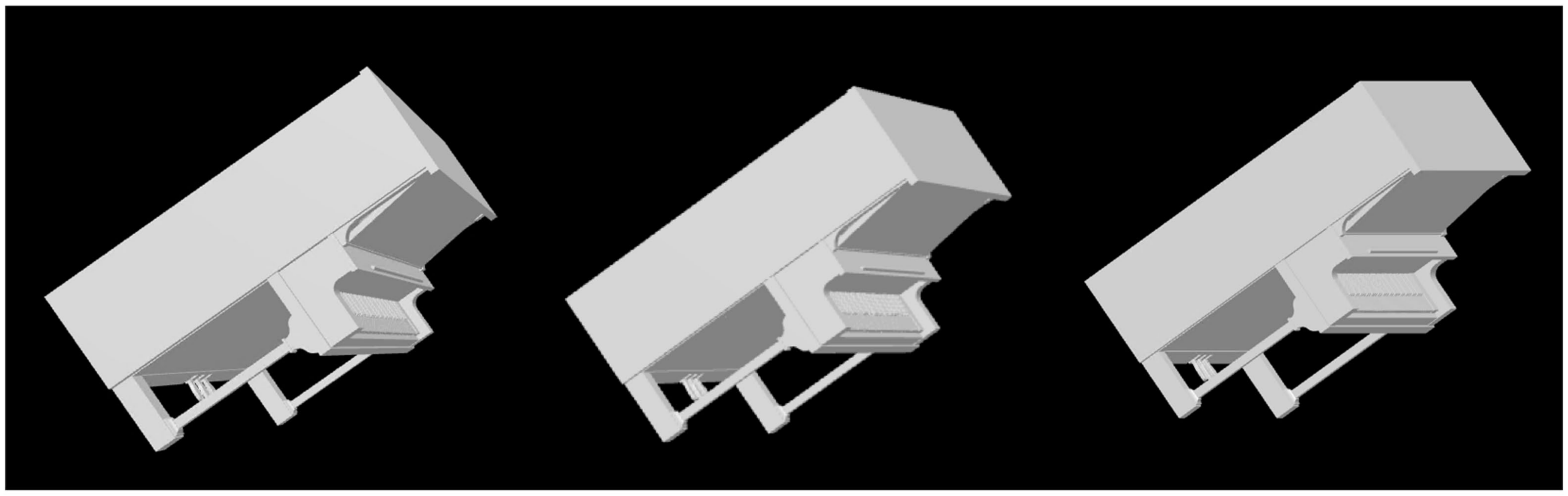
**(Left)** A perspective Image of an object under the viewing conditions in our experiment. Specifically, your viewing distance should be 2.4 times the diameter of this image. **(Center)** A perspective image of the same object at distance three times greater. We enlarged the size of this image to make the comparison of images easier. Your viewing distance should now be 7.2 times the diameter of this image, **(Right)** an orthographic image of the same object. All three images used the same slant and tilt of the 3D shape.

As illustrated in Figure 2, perspective images were larger (by a factor of about 3) than orthographic images. The reason for this difference is as follows. To make sure that a perspective image of an object is clearly different from an orthographic image of the same object, the range in depth of the 3D object, when perspective projection is used, must be non-negligible relative to viewing distance. So, moving a 3D object closer to the viewer (or to the camera) will increase perspective distortions as well as the size of the retinal (or camera) image. Figure 3 illustrates the perspective distortions of an object from our experiment in comparison to an orthographic image. We also show a perspective image of the same object when the viewing distance was increased by a factor of 3. We equated the sizes of these images for easier comparison.

The 3D orientation of the reference shape varied randomly from trial to trial. This orientation can be specified by the slant and tilt of its symmetry plane. On each trial, tilt (modulo 90 degrees) was constrained to lie between 15 and 75 degrees. Slant was constrained to be 15, 30, 45, 60, or 75 degrees. Each slant angle was used 20 times for a total of 100 trials per condition. On average, each condition took subjects about 45 min.

In each trial, the adjustable 3D shape was rotating around the x (horizontal) axis. None of the images of the rotating 3D shape was identical with the image of the reference 3D shape. This prevented the subject from matching 2D features during the adjustment. This was accomplished by applying a 3D rotation by 90 deg. around the y (vertical) axis to the reference shape before showing it as an adjustable shape. In addition, the size of the rotating 3D object was 80% of the size of the reference 3D object to encourage the subject to pay attention to shape, not size.

## Results

As explained in the methods section, a 2D orthographic image of a 3D symmetrical shape determines that 3D shape up to a single free parameter. The set of 3D shapes generated by different choices of this free parameter is termed the one parameter family. Two members of the one parameter family are different but related 3D shapes. In particular, one can be transformed into the other by stretching or compressing along two orthogonal directions. One of these directions is the normal of the symmetry plane. The other depends on the viewing direction that created the orthographic image. Each member of the one parameter family has a *unique* aspect ratio along these two orthogonal directions. Following Li et al. (2011) we define a measure of shape dissimilarity between two members of the one parameter family based on this *unique* aspect ratio [refer to Appendix B in Li et al. (2011) for details]. This shape dissimilarity measure will be referred to in this paper as *dissimilarity*. If dissimilarity is equal to x, then the aspect ratio of the shape recovered by the subject is $2^{x}$ times the aspect ratio of the reference 3D shape. A dissimilarity of zero implies that the subject recovered the reference shape veridically. A dissimilarity of −1 implies that the subject reconstructed a 3D shape which has aspect ratio one half that of the reference shape. In order to make the interpretation of the results clearer, let us define the shape dissimilarity in terms of the slants of the symmetry plane. Remember, the one parameter family can be described either by the aspect ratio of the shapes or by the slant of the symmetry plane of the shapes. These are two different parameterizations of the same one parameter family. Let σ be the slant of the symmetry plane of the reference 3D shape and $\sigma_{r}$ the slant of the symmetry plane of the 3D shape adjusted (reconstructed) by the subject. If the subject reconstructed the 3D shape veridically, $\sigma =\sigma_{r}\text{,}$ and shape dissimilarity is zero. Note that the adjustable 3D shape was constantly rotating and none of the 3D shapes of the rotating object had the same 3D orientation as the reference 3D object. The subjects in the experiment were matching the aspect ratios of the 3D shapes, not the slants of their symmetry planes. But once the adjustment is done, we can represent the shape dissimilarity by comparing the two slants from the same one parameter family. So, $\sigma_{r}$ refers to the adjusted 3D shape from the one parameter family defined by the orthographic image of the reference shape. Shape dissimilarity is a binary logarithm of $\tan\left(\sigma \right)/\tan\left(\sigma_{r}\right)$ . It follows that negative shape dissimilarity means that $\sigma_{r}>\sigma$ because tangent of an angle is a monotonically increasing function of the angle in (0, π/2).

**Figure 4:**
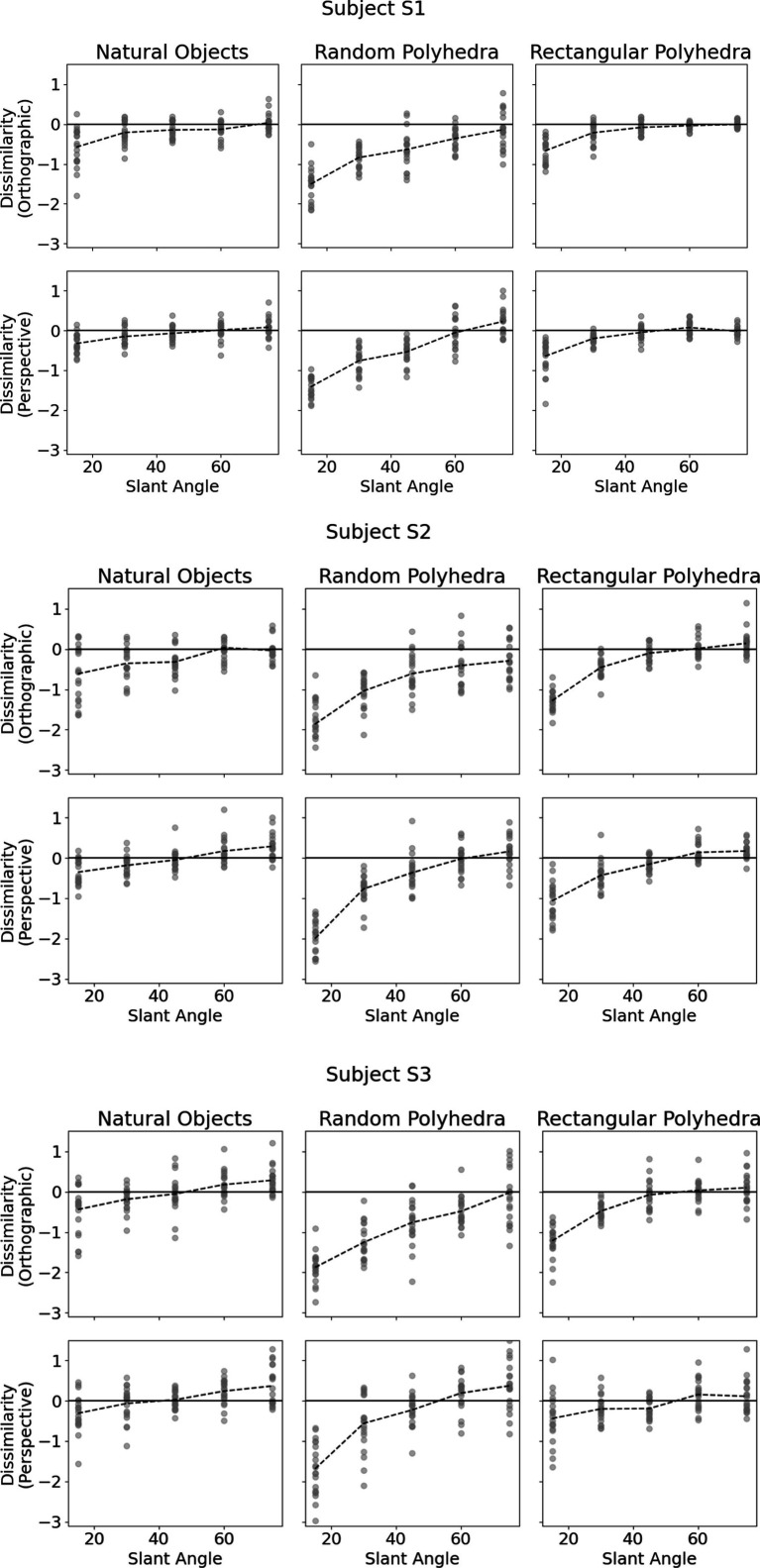
Subjects’ performance as a function of slant of the symmetry plane of the reference shape for each of the 6 conditions. The vertical axis denotes shape dissimilarity, while the horizontal axis denotes slant angle of the symmetry plane of the reference shape. Shape dissimilarity is the measure of how far the subject’s percept was from the reference shape. The data points represent the individual trials. The dashed line indicates average dissimilarity.

Figure 4 shows subjects’ performance in each of the six conditions. The vertical axis of each plot shows shape dissimilarity, while the horizontal axis shows the slant angle of the reference shape. A slant angle of 15 means that the viewing angle producing the image was such that the angular difference between image plane normal and symmetry plane normal was 15 degrees. In other words, the symmetry plane of the reference object was only 15 degrees off compared to the image plane. Li et al. (2011) used objects similar to the random polyhedra used in this experiment. They reported greater absolute values of shape dissimilarity for small slant angles than large slant angles. This pattern was reproduced in our experiment and holds for all types of objects. The most important result of our present experiment was that dissimilarity was much lower with natural objects and with rectangular polyhedra than with the random symmetrical polyhedra condition. This means that subjects were more accurate at reconstructing natural and rectangular objects than they were at reconstructing random polyhedra. Next, performance of our subjects with random polyhedra was similar to the monocular performance of subjects in Li et al.’s (2011) experiment. Our random polyhedra were not identical to the random polyhedra used by Li et al., but performance of our three subjects was very similar to the performance of the four subjects in Li et al.’s experiment. Finally, monocular performance of our subjects with natural objects and with rectangular polyhedra was almost as good as binocular performance of subjects in Li et al.’s experiment with random polyhedra. This result makes sense, intuitively. Objects appear to us the same regardless whether we look at them with one or two eyes. Apparently, natural objects have enough regularities (constraints) so that the ill-posed inverse problem of reconstructing their 3D shapes is solved nearly perfectly by our visual system. We will explain the nature of these constraints in the model section of our paper.

**Figure 5:**
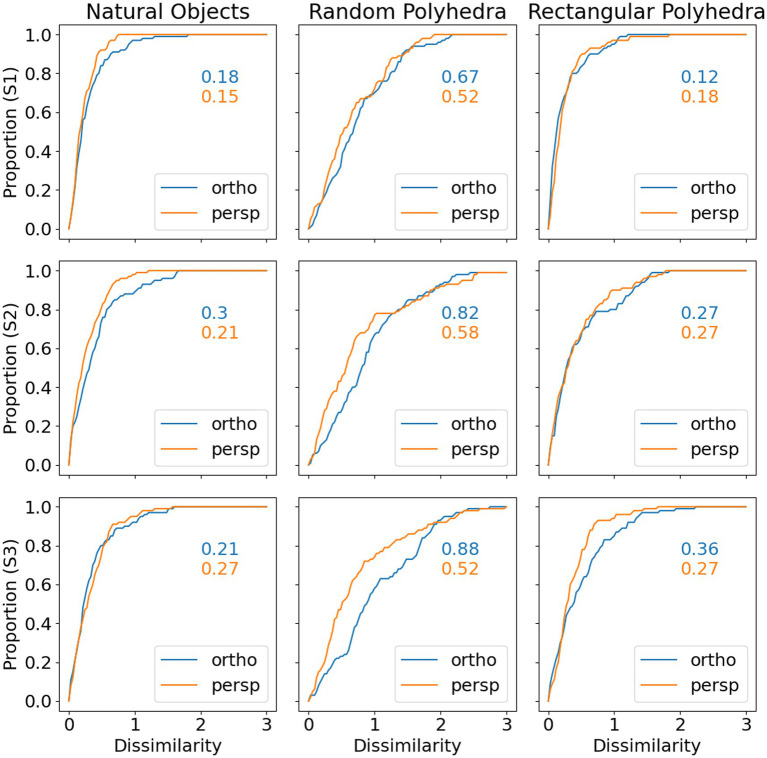
Cumulative distributions of absolute value of shape dissimilarity, by subject (rows) and object type (columns). The numbers inside the graphs are 50th percentiles.

**Figure 6:**
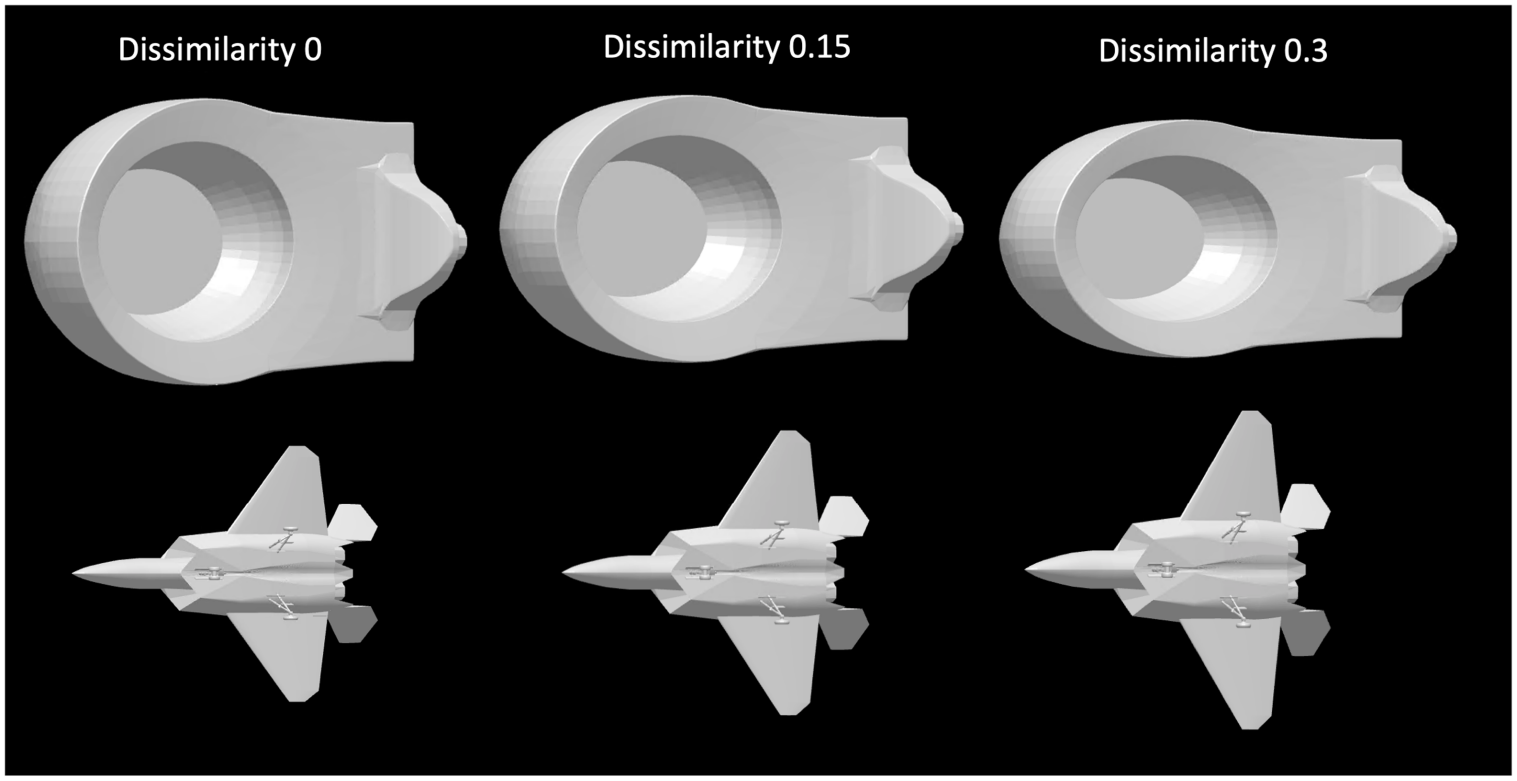
The leftmost column contains images of two reference shapes used in the experiment. The middle and right columns show images of shapes which have dissimilarities of 0.15 or 0.3 relative to the reference shape. A shape difference of 0.15 corresponds to a difference in aspect ratio of 11% while a shape difference of 0.3 corresponds to a difference in aspect ratio of 23%. The object in the top row has been stretched horizontally and compressed vertically. The object in the bottom row has been stretched vertically and compressed horizontally.

Next, we will discuss in some more detail the veridicality of the perceived shape and the difference between orthographic and perspective projection. Figure 5 shows cumulative distribution functions summarizing the data shown in Figure 4. Now, shape dissimilarity is on the horizontal axis. More precisely, the horizontal axis is the absolute value of shape dissimilarity. Let us denote this as AD. The vertical axis in Figure 5 shows the proportion of objects with absolute value of dissimilarity less than AD. In Figure 5, results from orthographic and perspective images are superimposed on the same graph. This allows a better comparison of these two experimental conditions. Figure 5 shows again that all subjects were much more accurate at recovering natural shapes and rectangular polyhedra than random symmetrical polyhedra. Performance with natural shapes was almost perfect. One way to illustrate this is to look at the 50th percentile of AD (these 50th percentiles are shown in each graph in Figure 5). The 50th percentile for natural shapes across the 3 subjects and two projections (orthographic and perspective) ranges between 0.15 and 0.3. This corresponds to errors in aspect ratio of the shape ranging between 11 and 23%. We can conclude that this performance is extremely good. Figure 6 shows examples of the difference in aspect ratio 11 and 23%. This shows that even though the reconstructed 3D shapes are not identical to the reference 3D shapes, the monocular percepts of our subjects are not far from veridical. We want to emphasize that we are evaluating veridicality of metric aspects of 3D shapes. To the best of our knowledge, this is the first result demonstrating veridicality of shape perception with natural objects. Subjects demonstrated a great degree of consistency in their reconstructions. Using slant as the dependent variable, the pairwise correlations between subjects while viewing natural shapes or rectangular polyhedra ranged from 0.91 to 0.97. Pairwise correlations between subjects while viewing random polyhedra were lower, ranging from 0.75 to 0.82.

Next, Figure 5 shows that reconstructions from perspective images tended to be more accurate than reconstructions from orthographic images and performance of all 3 subjects was very similar. S1 produced slightly more accurate and reliable results. This was not surprising considering the fact that S1 received more practice.

Finally, the graphs in Figure 5 show that subject performance with natural objects was similar to subject performance with rectangular polyhedra. We know from Perkins (1976) that rectangularity is likely a constraint used by the visual system. Rectangularity could explain high degrees of reconstruction accuracy on those natural objects which were rectangular. However, a number of the natural shapes are not rectangular and subjects performed well with these shapes also. This suggests that there are additional constraints employed by the visual system. This issue will be discussed in the next section.

## Model

### Symmetry correspondence

We formulated a computational model to emulate subjects’ performance with all three types of objects under both orthographic and perspective projection. The main question that we are trying to answer is which constraints are used by the human visual system. 3D mirror symmetry is the main constraint. In order to use mirror symmetry in 3D shape reconstruction the model has to know symmetry correspondence. Consider first the natural shapes. Given a 3D mesh, primary and secondary symmetry planes are estimated using RANSAC (Fischler and Bolles, 1981). Two random points are sampled and the unique symmetry plane bisecting the vector between them is computed. Next, the set of points in the mesh which are symmetric about this symmetry plane to within a certain tolerance is identified. Repeating this procedure N times yields N candidate symmetry planes and N sets of correspondences. The primary symmetry plane, $\pi_{1}$ , is the plane with the greatest number of correspondences. The secondary symmetry plane, $\pi_{2}$ , is defined as the plane (i) whose normal forms the angle equal to or greater than 45 degrees with the normal of the primary symmetry plane and (ii) whose correspondences overlap the most with the correspondences of the primary symmetry plane. By “overlapping correspondences” we mean triplets of points such that two points are corresponding with respect to one symmetry plane and two are corresponding with respect to the second symmetry plane. This procedure yields consistent estimates of primary and secondary symmetry planes so long as N is sufficiently large. In almost all cases, the two symmetry planes were orthogonal to each other. Next, consider the random symmetrical polyhedra in our experiment. These objects had only one plane of symmetry. The secondary plane was identified as the best estimate using large tolerance for symmetry correspondence. Finally, with the rectangular symmetrical polyhedra consisting of two rectangular boxes, two symmetry planes were always well defined. The primary symmetry plane was the symmetry plane of the entire object, and the secondary symmetry plane was one of the two additional symmetry planes of one of the rectangular boxes.

### Differences in the input received by model versus subject

Note that the model received more information than subjects. On each trial, the subject was shown a rendered image of a 3D opaque reference object. The model, on the other hand, was provided the (x, y) coordinates of the object points in the image assuming that the reference object was transparent. In addition, the model was provided the two sets of symmetry correspondences. In this work, we are not trying to explain how 3D symmetry correspondence is solved in a single 2D image, nor how the back invisible part of the object is reconstructed. This paper focuses on the nature of constraints that can account for near-veridical perception of our subjects. The two aspects of the problem, establishing symmetry correspondence and reconstructing the back, invisible part of an objects have been addressed, at least partially, in our prior work (Pizlo et al., 2014; Sawada et al., 2014).

### Image correction

The model forms a one-parameter family of 3D shapes consistent with an orthographic image of the 3D reference shape. However, the model is given a noisy version of a 2D orthographic or perspective image of the shape to emulate the noise in the human visual system. For every pair of image points that are projections of mirror symmetrical 3D points, we perturbed the orientation of the line segment connecting the image points by a random number generated from a normal distribution whose expected value was zero and standard deviation was 1 deg. This amount of noise is consistent with known difference threshold of line orientation discrimination (Orban et al., 1984). Once the model is given such a noisy 2D orthographic or perspective image, the model must begin with correcting the image before the one-parameter family is generated. As a result of this correction, all symmetry lines in the image become parallel. Recall that the one parameter family is defined only for a noiseless orthographic image. In the 3D symmetrical shape, the symmetry line segments are parallel and perpendicular to the symmetry plane. This parallelism is preserved under orthographic projection, but is not preserved under perspective projection or when noise is added to the image. When given a noisy perspective or orthographic image, the image must be corrected in order to become a valid orthographic image of a symmetrical 3D shape, which enables the generation of the one parameter family. The model makes all symmetry line segments parallel to each other in the least-squares sense (see Sawada, 2010).

### Constraints

Once the one parameter family of 3D symmetrical shapes consistent with the corrected image is generated, the model’s task is to select a single member from the one parameter family. This is accomplished by using constraints. Li et al. (2009) discussed a variety of constraints based on the idea of maximum compactness. Compactness, based on the volume and surface area of an object is defined in Equation 1. In practice, it may be difficult to calculate the volume and surface area of an object. For example, the wings of a bird have some volume, but the estimation of the volume will never be reliable. Therefore, it is preferable to compute compactness of the convex hull of an object. In all objects that we tested, there was a single member O* of the one parameter family which maximizes C_1_(O) of the convex hull of the object and therefore, maximum compactness is a valid constraint (selection rule). The maximally compact 3D shape is a sphere (Pólya and Szegö, 1951). As an object becomes increasingly elongated, compactness decreases. The maximum compactness rule encodes the idea that the 3D reconstruction should not be overly stretched out. Because the reconstructed 3D shape must be consistent with the 2D retinal image, the uncertainty about the stretch of an object refers to the uncertainty about the range of the object in depth direction. We discovered in our simulations that maximum compactness leads to what Leclerc and Fischler (1992) called “consistency criterion,” which is conceptually similar to shape constancy (see Appendix).

Li et al. (2009) also considered modified versions of compactness, $V{\left(O\right)}^{n}/S{\left(O\right)}^{m}$ , and found that subject’s performance was best replicated when *n* = 1, *m* = 3, as shown in Equation 2. Our simulations showed that the constraint represented by maximum of C_2_ is highly correlated with minimizing the range of the 3D object in depth direction (see Appendix).


(1) $$C_{1}\left(O\right)=\frac{V{\left(O\right)}^{2}}{S{\left(O\right)}^{3}}$$

(2) $$C_{2}\left(O\right)=\frac{V\left(O\right)}{S{\left(O\right)}^{3}}$$

**Figure 7:**
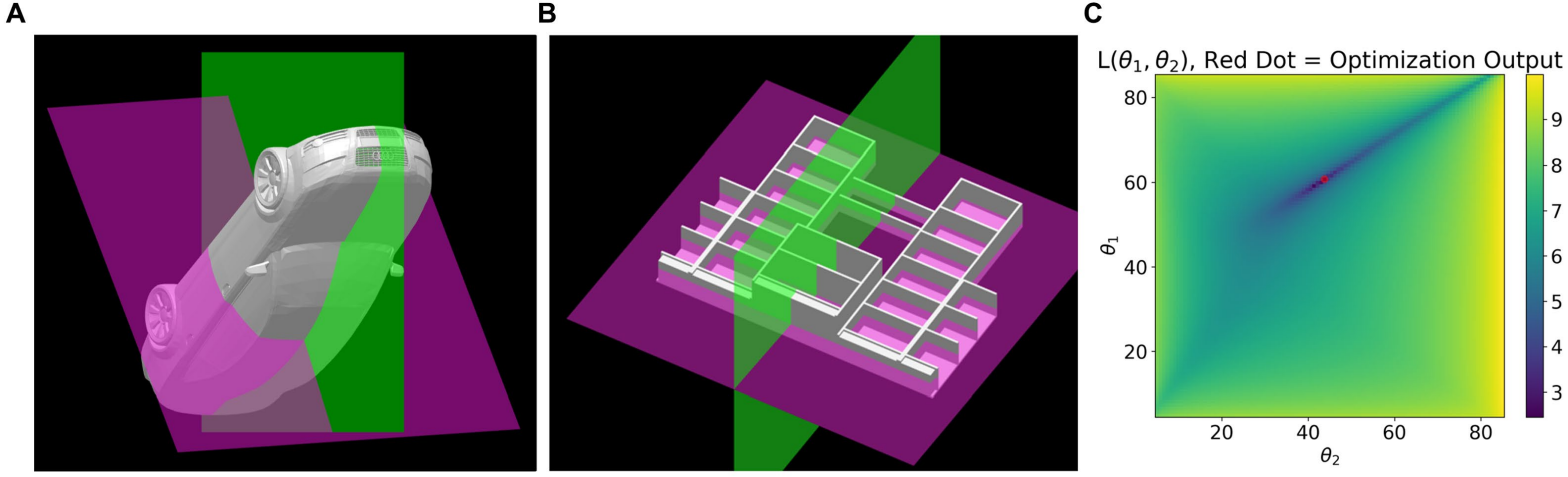
**(A)** and **(B)** each show two partial symmetry planes detected in 3D models. Both models are globally mirror symmetrical about the green plane and both have parts symmetrical about both green and purple planes. For example, the wheels of the car are symmetrical about both green and purple planes. Plot **(C)** shows the cost surface defined in Equation 3 for the car shown in **(A)**. Dark blue corresponds to points close to the minimum and the red dot is the global minimum.

We also used an additional constraint based on the two symmetry planes described earlier. Many objects which are globally mirror symmetrical have parts which are approximately mirror symmetrical about two planes of symmetry. See Figure 7. Two planes of mirror symmetry produce two sets of symmetry correspondences and therefore two one-parameter families. If the images are orthographic and noiseless, the subset of points shared by the two one parameter families will perfectly overlap at the true shape, up to a constant shift in depth. With a corrected noisy image, the two one parameter families will have members which are “closest” to each other. The selection rule (constraint) $C_{3}\left(O\right)$ selects the member of the one parameter family associated with the primary symmetry plane which is closest to a member of the one parameter family associated with a secondary symmetry plane. A metric defining the distance between two symmetrical 3D shapes associated with the corrected images is defined in Equation 3. This equation is described next.

Each set of correspondences of a one parameter family has a symmetry plane $\pi_{k}$ at slant $\theta_{k}$ , where $\theta_{k}$ can range from zero to ninety degrees. Different choices of slant will yield different depth values. A loss function L over depth values generated by the two one parameter families is shown in Equation 3. In all cases that we tested, L generated a unique minimum. Minimizing L corresponds to finding depth values for parts of an object such that those parts are maximally symmetrical about two planes of symmetry in 3D. The selection rule $C_{3}\left(O\right)$ returns slant angle $\theta_{1}^{*}$ which is the slant of the primary symmetry where L is minimal, for some $\theta_{2}^{*}\text{.}$


(3) $$\begin{array}{l} \frac{\;}{z\theta_{k}}=\frac{1}{N}\overset{N}{\underset{i=1}{\sum }}z_{\theta_{k},i}\\ L\left(\theta_{1},\theta_{2}\right)=\overset{N}{\underset{i=1}{\sum }}{\left[\left(z_{\theta_{1},i}-\bar{z_{\theta_{1}}}\right)-\left(z_{\theta_{2},i}-\bar{z_{\theta_{2}}}\right)\right]}^{2} \end{array}$$

In Equation 3 $z_{\theta_{k},i}$ is the depth value associated with vertex i from symmetry plane $\pi_{k}\text{.}$ Ideally, the depth value from symmetry plane $\pi_{1}$ will be the same as the depth value from $\pi_{2}\text{.}$ Parameters $\theta_{1},\theta_{2}$ are slants of symmetry planes $\pi_{1},\pi_{2}$ .

The three constraints, C_1_, C_2_ and C_3_, each select a member of the one parameter family of a corrected image. If present, the perspective information in an image is also useful in choosing the unique 3D shape from the one parameter family of shapes. A perspective image of a 3D symmetrical shape allows for unique reconstruction of that symmetrical shape. This is achieved by relating the vanishing point of the symmetry line segments to the slant of the symmetry plane as described by Sawada et al. (2011). The vanishing point provides the fourth estimator of the slant of the symmetry plane as follows. We consider all pairs of symmetry line segments in the 2D image and estimate the vanishing point for each pair of symmetry lines as the intersection of these lines. The vector connecting the center of perspective projection of the camera used by the model and the vanishing point is normal to the symmetry plane of the 3D shape. As a result, from each pair of symmetry lines we obtain an estimate of the slant of the symmetry plane. We then take the median of these. Note that in an orthographic image, parallelism of symmetry line segments is preserved so the vanishing point always lies at infinity. It follows that the estimated slant of the symmetry plane is 90 deg. in such a case. The estimate of slant from perspective information is likely to be wrong when an orthographic image is used and so, the weight of this estimate should be close to zero. But when the 2D image is a perspective image, the vanishing point does provide a useful estimate and its weight should be positive. Therefore, any model which seeks to use perspective information needs some way to quantify the reliability of perspective information in the 2D image.

### Model definition

For each trial, we have four point estimates of slant of the primary symmetry plane. We combine these estimates using the model shown in Equations 4, 5. In this model, the slant predicted by perspective information is given weight λ , and the slant predicted by constraints C_1_, C_2_ and C_3_ is given weight 1−λ .


(4) $$\hat{\theta }=\lambda \theta_{\mathrm{persp}}+\left(1-\lambda \right)\theta_{LM}$$

(5) $$\theta_{LM}=\beta_{0}+\beta_{0}+\beta_{1}\theta_{v2s3}+\beta_{2}\theta_{s3v}+\beta_{3}\theta_{sym2}$$

**Figure 8:**
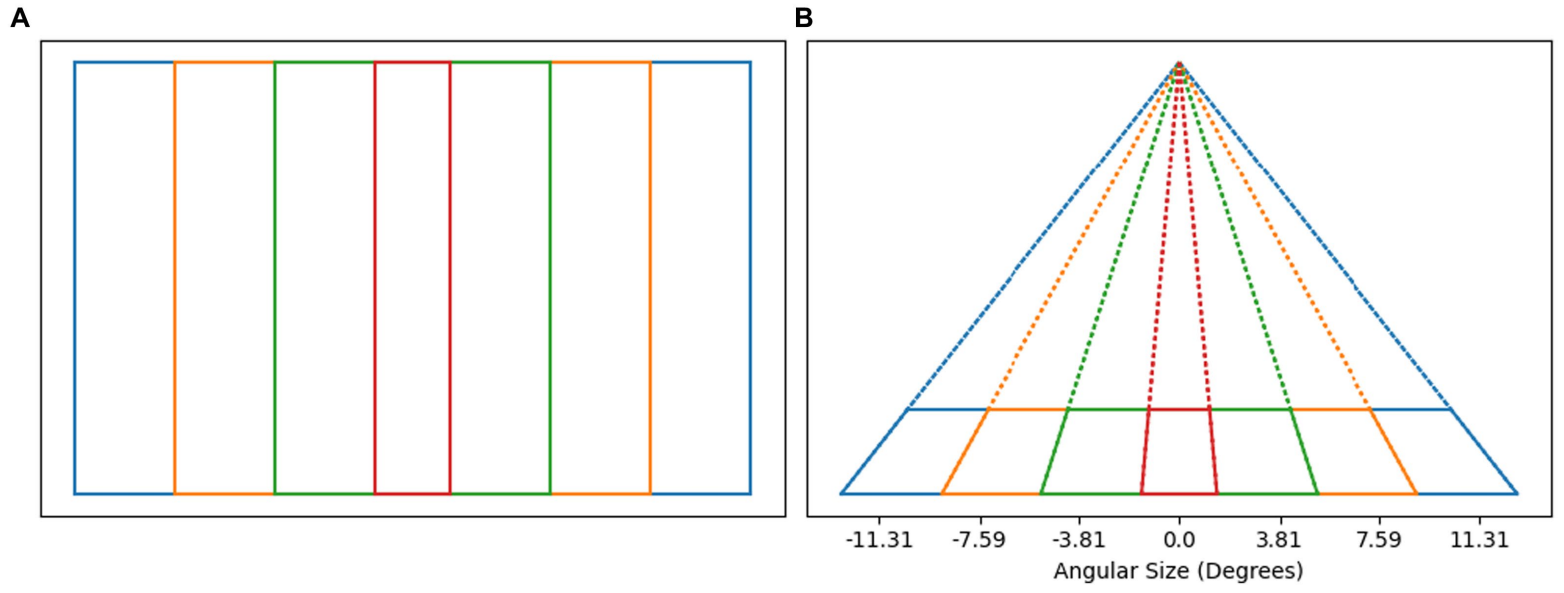
**(A)** Shows a set of rectangles of equal height in a plane parallel to the image plane. If we treat the vertical sides of the rectangles as symmetry lines in 3D, then the slant of the symmetry plane is 90 deg. – the symmetry plan is orthogonal to the image. **(B)** Shows the perspective images of those rectangles (solid lines) after rotating the rectangles by 75 deg. away from the frontoparallel plane. In this case, the slant of the symmetry plane is 15 deg. Note that the angular width of the perspective image is related to the angle between symmetry line segments in the image. The angular size of orthographic images in our experiment was around 7 degrees, corresponding to −3.5 to 3.5 on the plot. The angular size of perspective images in our experiment was around 20 degrees, corresponding to −10 to 10 on the plot. Perspective images in our experiment had strong perspective information.

The weight given to perspective estimate of slant should be related to the reliability of perspective information. As an estimate of the reliability of perspective information, we use a function of the angular size of the image. Conventionally, the degree of perspective distortion has been related to the range in depth of the object relative to the viewing distance. For any 3D object, the ratio of range in depth to viewing distance will increase as the object is brought closer to the observer. The image size will also increase as the object is brought closer to the observer. It turns out that the image size is also correlated with degree of perspective distortion. To quantify the strength of perspective distortion in the image, consider Figure 8. Figure 8A shows four rectangles in a plane parallel to the image plane. All four rectangles have identical height but different widths. If the vertical sides of these rectangles are treated as symmetry lines in 3D, the symmetry plane is orthogonal to the image and has slant 90 deg. Figure 8B shows a perspective image of these rectangles after these rectangles were rotated 75 degrees about their bottom edge. This results in the slant of the symmetry plane of the rectangles being 15 degrees. The vanishing point is easier to estimate if the angle between the symmetry line segments is larger. The formula for the angle between the symmetry line segments, ϕ, is given in Equation 7. Note that this equation is a function of the tangent of the angular image size gamma (Equation 6). In this equation there is a free parameter, t, corresponding to the degree of rotation about the horizontal axis the rectangle. We used *t* = 75 deg. because it led to the best fit of the model to the subjects’ data. Finally, the weight λ (Equation 8) of perspective information is defined as a monotonically increasing function over the range (0, π) that maps the range of ϕ to [0,1]. This results in a model for which perspective information is weighted more as the angular size of the image increases. The weight assigned to perspective information is grounded in the geometry of the perspective image, rather than in the geometry of the 3D reconstructed shape.


(6) $$\alpha =\tan\left(\gamma /2\right)$$

(7) $$\phi =2\tan^{-1}\left(\alpha \tan\left(t\right)\right)$$

(8) $$\lambda =\frac{1-\cos\left(\phi \right)}{2}$$

Coefficients β_0_ – β_3_ in the linear model in Equation 5 are selected to minimize the sum of squared errors between subject slants and model slants. We fit a linear model separately for each of the 3 types of shapes: natural objects, random symmetrical polyhedra and rectangular symmetrical polyhedra composed of two rectangular boxes. It was natural to expect different coefficients across these three object types. For example, the constraint produced from two symmetry planes was expected to have large weight with natural objects and rectangular polyhedra, but not with random polyhedra that had only one symmetry plane. This indeed was the case. The model was fit to each individual subject to account for individual differences. We want to emphasize, however, that we used the same model for orthographic and for perspective images. The model’s reconstructions were different when orthographic vs. perspective images were used because the contribution of θ_persp_ was modulated by the parameter λ that represented the reliability of perspective information.

### Model results

Recall from our psychophysical experiment that subjects’ performance with random polyhedra was different than with rectangular polyhedra and with natural objects. Also, subject S1 produced more accurate reconstructions than the other two subjects. In this section, we report model fit for every combination of subject and object type (Natural Objects, Random Polyhedra, Rectangular Polyhedra).

**Figure 9:**
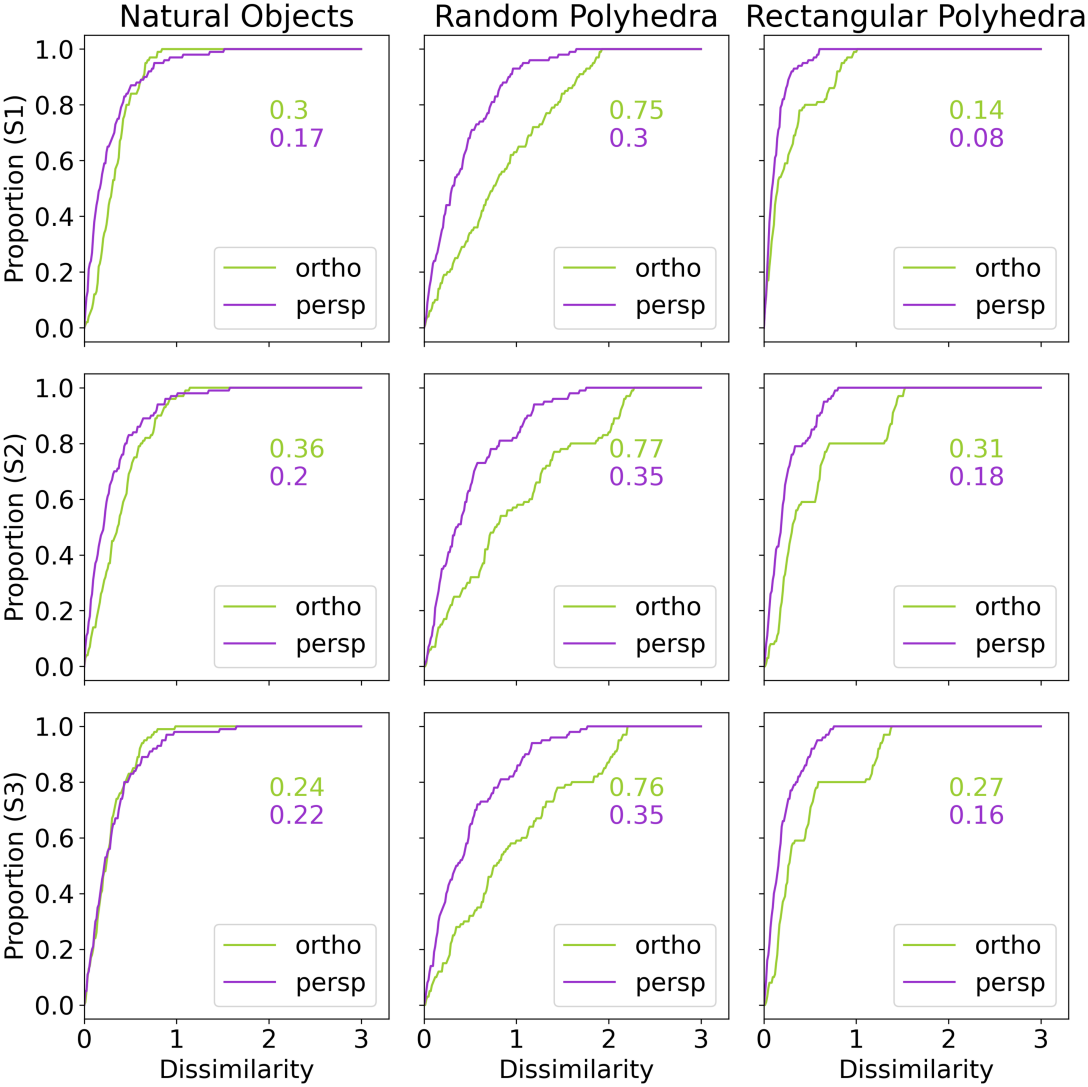
Cumulative distributions of dissimilarity between model reconstruction and true shape, by subject and condition. The numbers inside the graphs are 50th percentiles.

In Figure 9 we show cumulative functions for the model. Figure 9 is directly comparable to Figure 5, but where Figure 5 compared the subjects’ reconstructions to the true shapes, Figure 9 compares the model reconstructions to the true shapes. Figures 5, 9 show similar median shape dissimilarities. Also, the model shows better performance with perspective images compared to orthographic images.

**Figure 10:**
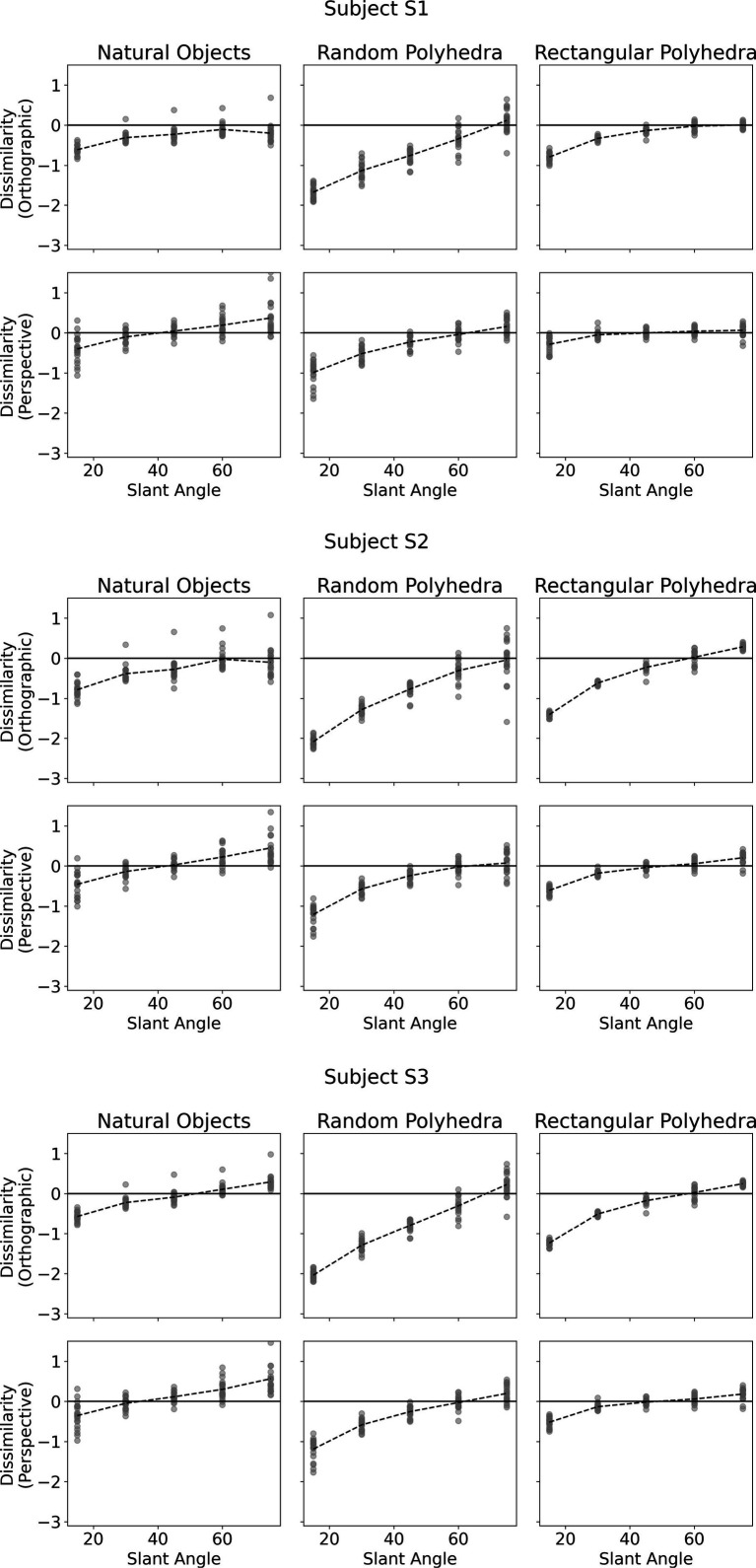
Dissimilarities between true shape and model shape, for all three subjects. Note that in some panels there is less variability in the model than in subject’s responses which are plotted in Figure 4.

There appear to be discrete steps present in some of the cumulative functions of Figure 9, particularly for rectangular polyhedra. These step functions can be explained by considering Figure 10, which directly shows the shape dissimilarities between true shape and model shape. Some of the panels in Figure 10 show that the model’s predictions are clustered around the mean for individual veridical slants. This tight clustering can result in steps when the dissimilarities are replotted as a cumulative curve. The plots in Figure 10 are directly comparable to the plots in Figure 4. The mean subject performance shown in Figure 4 is reproduced well by the model, but the model tends to have lower variability.

**Figure 11:**
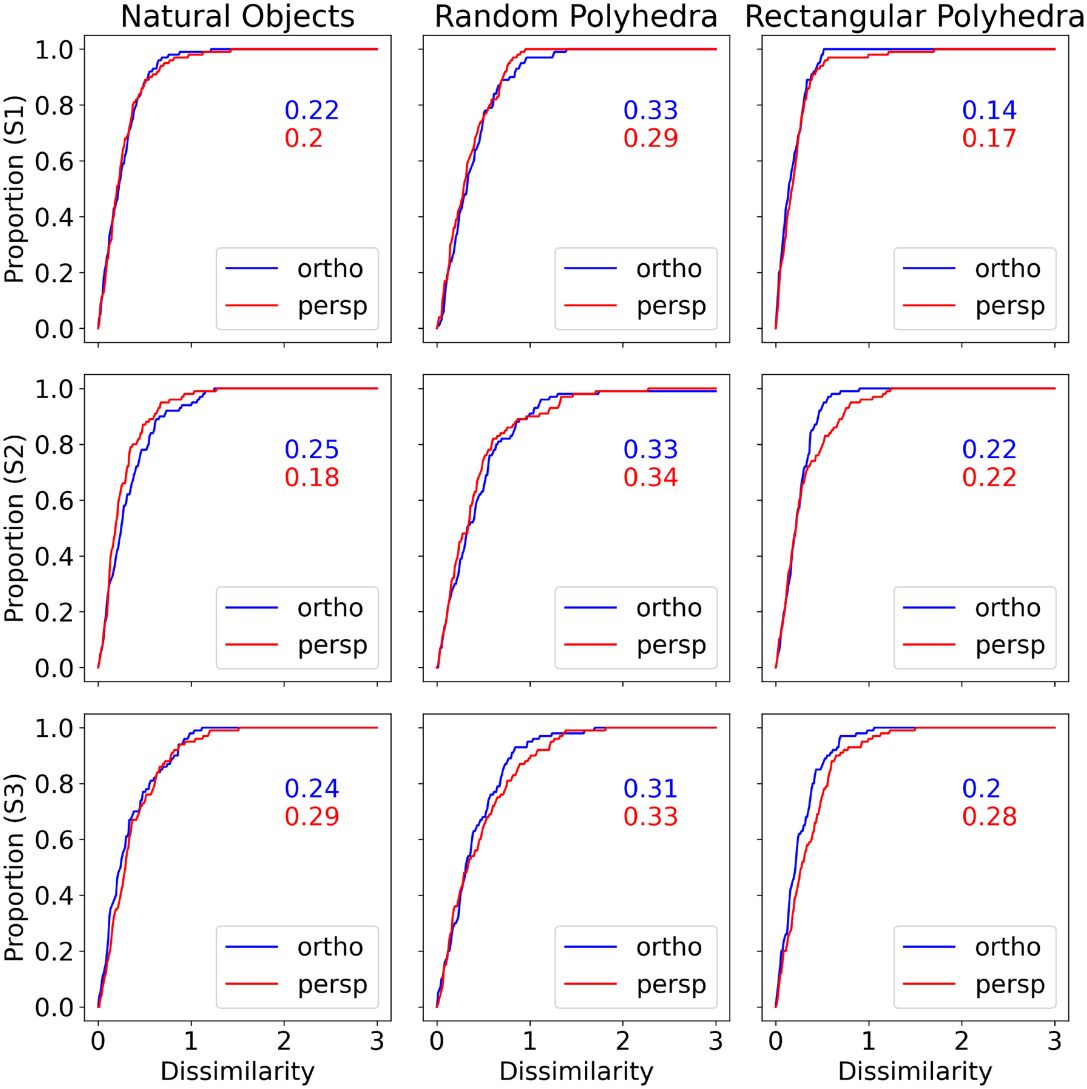
Cumulative distributions of dissimilarity between subject reconstruction and model reconstruction, by subject and condition. For all object types and subjects, the curves for perspective and orthographic images are similar, indicating that the model captured subject percept on perspective and orthographic images equally well. The median shape differences tend to be around 0.3, corresponding to the model predicting an aspect ratio that was 23% different from the aspect ratio selected by the subject.

Figure 11 shows cumulative plots for the dissimilarities between shapes reconstructed by the model and shapes reconstructed by subjects. These graphs represent direct comparison between the subject’s reconstructed shape and model’s reconstructed shape. Recall that the subjects and the models were tested with the same 3D shapes shown at the same 3D orientations. For each trial the aspect ratio of the reconstructed shape by the subject was compared to the aspect ratio of the reconstructed shape by the model. The median shape difference between subject and model is around 0.3, for all combinations of subject, object type and projection type. A shape dissimilarity of 0.3 corresponds to the model reconstructing a shape with an aspect ratio which is 23% different compared to the aspect ratio selected by the subject. To further evaluate the similarity between the model and subject’s reconstructions, we computed correlation coefficients between the slants reconstructed by the subject and the slants reconstructed by the model, the same way we computed correlations between pairs of subjects when we described our psychophysical results. The pairwise correlations between the subject and the model while viewing natural shapes or rectangular polyhedra ranged from 0.92 to 0.98. Pairwise correlations between the subject and the model while viewing random symmetrical polyhedra were lower, ranging from 0.74 to 0.93. These correlations are almost identical to the pairwise correlations between subjects.

## Summary and discussion

Previous studies have explored monocular 3D perception of many types of objects (polyhedra, objects composed of geons, planar symmetrical figures and highly irregular shapes), but not natural objects. To our knowledge, this is the first study on monocular 3D perception of natural objects, and our results suggest that monocular perception of natural objects is very accurate. Our shape reconstruction experiment considered perspective and orthographic images of natural objects, random symmetrical polyhedra and rectangular polyhedra. We observed a marked decrease in reconstruction accuracy with random symmetrical polyhedra compared to the other categories. We also observed a slight increase in reconstruction accuracy with perspective, compared to orthographic images.

These results can only be explained by the application of constraints by the human visual system. We modelled subject’s performance on orthographic and perspective images, employing mirror symmetry and compactness constraints. Specifically, we employed an implicit constraint of mirror symmetry and explicit constraints of compactness, modified compactness and a search for parts of objects which have two planes of symmetry. We also added an estimate of the slant based on the vanishing point when perspective information was available. Each of these constraints generated an estimate of 3D shape, parameterized by the slant of its symmetry plane. The slants from the four estimates were combined using a linear model fit to subject data. Putting these four estimates together allowed us to fit subject responses fairly accurately for all three object types and both types of projection.

One of the reviewers asked about possible extensions of the model to binocular viewing and viewing of a rotating object. The case of binocular viewing was examined in Li et al. (2011). In that model, the binocular depth order was used as an additional constraint in reconstructing the 3D shape. The same can be done with our new model. When a rotating 3D shape is shown, the 3D reconstruction can be improved by adding a 3D rigidity constraint in a way analogous to Ullman’s (1984) maximizing rigidity algorithm worked. We will address both these extensions in our future work.

To conclude, the human visual system is remarkable in its ability to accurately reconstruct 3D natural objects. In our experiment with perspective and orthographic images, the median error between reconstructed aspect ratio and true aspect ratio ranged between 11 and 23% with natural objects. Subjects are familiar with airplanes, cars, guitars and toilets, but are they sufficiently familiar with, say, airplanes to reconstruct a particular airplane’s aspect ratio to within 23% of its true value? We believe there is much more to 3D perception than familiarity. An explainable and interpretable model of the visual system must combine knowledge of projective geometry with constraints used by the visual system, as revealed by psychophysical experiments. The fact that the model proposed here produces reconstructions that are highly correlated with human reconstructions strongly suggests that we have identified the most important 3D shape constraints that are used by the human visual system. The remaining challenge is to explain how the visual system establishes 3D symmetry correspondence in a single 2D skew-symmetrical image.

## Data availability statement

The raw data supporting the conclusions of this article will be made available by the authors, without undue reservation.

## Ethics statement

The studies involving humans were approved by UCI Institutional Review Board as a self-determined exemption. The studies were conducted in accordance with the local legislation and institutional requirements. The participants provided their verbal informed consent to participate in this study.

## Author contributions

MB: Conceptualization, Formal Analysis, Investigation, Writing – original draft, Writing – review & editing. ZP: Conceptualization, Supervision, Writing – original draft, Writing – review & editing.

## Figures and Tables

**Figure 12:**
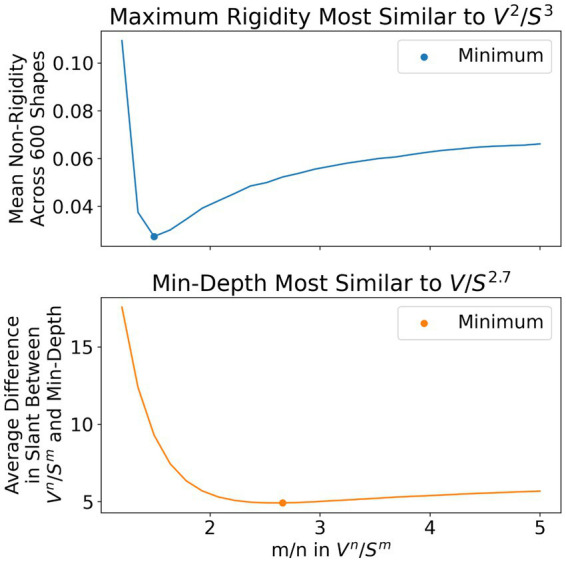
We used a range of cost functions of the type $V{\left(O\right)}^{n}/S{\left(O\right)}^{m}\text{.}$ This plot shows that $V{\left(O\right)}^{2}/S{\left(O\right)}^{3}$ produces the most rigid (consistent) reconstructions, while $V\left(O\right)/S{\left(O\right)}^{3}$ produces reconstructions that are closest to the minimum range in depth. The specific distance measures used on the vertical axes of the plots are described in the main body of text.

## Appendix

Our work emphasizes the role of symmetry and compactness as constraints the visual system imposes in order to arrive at a unique and accurate 3D reconstruction from a single image. Leclerc and Fischler (1992) considered the same problem and introduced the idea of a “consistency criterion” for deciding whether one reconstruction model was superior to another. The “consistency criterion” is the idea that the best model (shape constraint) is the model which generates the most consistent 3D reconstructions across many different views of an object. In other words, whatever constraints the model uses, the model which produces the most shape constancy is the best model. In Figure 12, we considered many models which selected a member of the one parameter family of 3D symmetrical shapes by maximizing the ratio $V{\left(O\right)}^{n}/S{\left(O\right)}^{m}$ . We considered the performance of these various models in a simulation experiment based on the same 600 objects used in the experiment. Specifically, we used 200 random symmetrical polyhedra, 200 rectangular symmetrical polyhedra and 200 natural objects. Orthographic images of these objects were taken at identical viewpoints to those in the experiment. Given an m, n pair and an orthographic image of one of the 600 objects, we selected a member of the one parameter family maximizing $V{\left(O\right)}^{n}/S{\left(O\right)}^{m}$ . We then rotated the 3D reconstructed shape by 7.5 degrees about the y-axis, took an orthographic image, generated a new one parameter family based on the new image, selected a member of this new one parameter family using the same m, n pair and finally, computed a difference (non-rigidity) between the two reconstructions. This non-rigidity was computed as follows. Both 3D reconstructions could have generated the second orthographic image and differ only in the depth they assign to points in this second orthographic image. The distance measure we used was the average absolute difference in depth between points in one reconstruction to the corresponding points in the other reconstruction, after both reconstructions have been shifted to the same mean depth. A distance of zero corresponds to perfectly rigid reconstructions. Averaging over all 600 images (one image per object) for a particular choice of m, n yields the first plot in Figure 12. This first plot shows that constraint C_1_ produces the most rigid (consistent) 3D reconstructions. Similar results were produced for rotations other than 7.5 deg.

The second plot in Figure 12 illustrates the minimum range in depth constraint. Here, we selected the member of the one parameter family which had minimal range in depth. This second plot shows that constraint $V\left(O\right)/S{\left(O\right)}^{3}$ produces results that are similar to the minimum range in depth constraint. The vertical axis in this plot is the difference between the slant selected by constraint $V{\left(O\right)}^{n}/S{\left(O\right)}^{m}$ and the slant selected by the minimum depth constraint, averaged across all 600 trials. The second plot shows that, on average, the slant selected by minimum depth constraint is most similar to the slant selected by $V\left(O\right)/S{\left(O\right)}^{2.7}$ .
